# Fallbericht zum traumatischen Totalabriss der Trachea vom Kehlkopf mit positivem Ausgang

**DOI:** 10.1007/s00106-022-01159-9

**Published:** 2022-04-01

**Authors:** Ulrich Kisser, Friedemann Pabst, Sylva Bartel, Dominik Schramm, Alexander Glien, Stefan K. Plontke, Daniel Ebert, Jan Wittlinger

**Affiliations:** 1 Universitätsklinik für Hals-Nasen-Ohrenheilkunde, Kopf- und Halschirurgie, Universitätsklinikum Halle (Saale), Ernst-Grube-Str. 40, 06120 Halle (Saale), Deutschland; 2 Klinik für Hals-Nasen-Ohrenheilkunde, Kopf-Hals-Chirurgie, Plastische Operationen, Städtisches Klinikum Dresden, Dresden, Deutschland; 3 Department für Strahlenmedizin, Klinik und Poliklinik für , Radiologie, Universitätsklinikum Halle (Saale), Halle (Saale), Deutschland; 4 Klinik für Anästhesie und Operative Intensivmedizin, Universitätsklinikum Halle (Saale), Halle (Saale), Deutschland

**Keywords:** Halsverletzungen, Ruptur, Unfallverletzungen, N.-laryngeus-recurrens-Trauma, Atemwegskrankheiten, Neck injuries, Rupture, Accidental injuries, Recurrent laryngeal nerve trauma, Respiratory tract diseases

## Abstract

**Anamnese:**

Eine 21-jährige Patientin erlitt im Rahmen eines Unfalls ein schweres Strangulationstrauma. Stridor und Dyspnoe setzten erst mit Verzögerung ein und führten zur Notfallintubation.

**Befund:**

Im Rahmen der klinischen Untersuchung zeigten sich Strangulationsmarken und ein Emphysem der Halsweichteile. Die Computertomographie erhärtete den Verdacht auf einen Abriss der Trachea vom Kehlkopf und ergab eine Fehllage des Beatmungstubus.

**Diagnose:**

Bei der weiteren chirurgischen Exploration zeigte sich eine komplette laryngotracheale (krikotracheale) Separation.

**Therapie und Verlauf:**

Nach initialer Nottracheotomie wurden in einem zweizeitigen operativen Verfahren die krikotracheale Reanastomosierung und die Retracheostomie durchgeführt.

**Schlussfolgerung:**

Laryngotracheale Separationen stellen den höchsten Schweregrad der Kehlkopfverletzungen dar und sind mit einer hohen Mortalität behaftet. Im geschilderten Fall überlebte die Patientin und konnte, trotz beidseitiger Rekurrensparese, dekanüliert entlassen werden.

## Abstract (en)

**History:**

A 21-year-old female was injured by accidental strangulation. Dyspnea and stridor occurred with delay, and led to emergency intubation.

**Findings:**

Physical examination showed strangulation marks and neck emphysema. Computed tomography confirmed laryngotracheal separation and revealed misplacement of the ventilation tube.

**Diagnosis:**

Further surgical exploration revealed complete laryngotracheal (cricotracheal) separation.

**Treatment:**

After initial emergency tracheotomy, cricotracheal reanastomosis was achieved by a two-stage surgical approach.

**Conclusion:**

Laryngotracheal separation is associated with high mortality. In the case presented herein, the patient survived and was discharged from hospital without a tracheostomy tube despite bilateral recurrent laryngeal nerve palsy.

## Falldarstellung

### Anamnese

Der Schal einer 21-jährigen Patientin war bei einer Feierlichkeit in einer Werkstatt in das mit hoher Geschwindigkeit rotierende Schneckengetriebe einer Kraftfahrzeug-Hebebühne geraten. Die Anwesenden konnten die Betroffene nach wenigen Sekunden befreien, ein Strangulationstrauma aber nicht verhindern. Der Notarzt wurde verständigt. Die Patientin konnte sich noch zu Fuß zum Rettungswagen begeben, entwickelte dann aber einen in- und exspiratorischen Stridor, woraufhin sie notfallmäßig intubiert und in den Schockraum der interdisziplinären Notaufnahme des Universitätsklinikums Halle gebracht wurde.

### Befund

**Figure 1:**
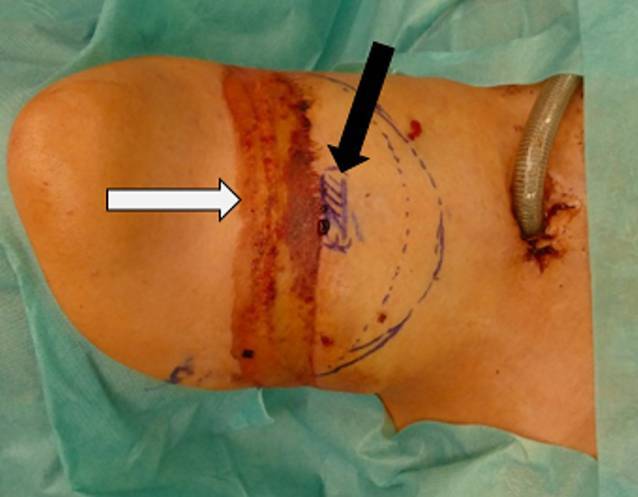

**Figure 2:**
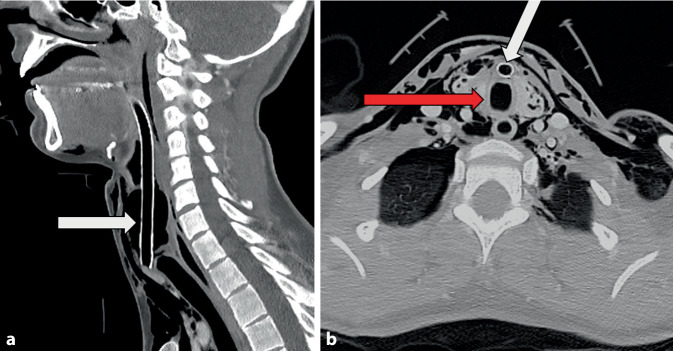

Bei der Inspektion zeigten sich äußerlich deutliche Strangulationsmarken am Hals (Abb. 1). Palpatorisch ließ sich ein ausgeprägtes Emphysem der Halsweichteile feststellen. Eine Computertomographie (CT, Traumaspirale) ergab den Verdacht auf einen vollständigen Abriss der Trachea vom Kehlkopf sowie ein ausgeprägtes zervikales und mediastinales Emphysem. Außerdem fiel auf, dass der Beatmungstubus ventral in den Halsweichteilen und nicht im Lumen der Trachea endete (Abb. 2). Darüber hinaus zeigte sich in der CT, dass Anteile der Trachealwand in das Lumen des distalen Trachealstumpfs hineinragten. Ein Anhalt für eine Verletzung des Ösophagus oder der Halsgefäße ergab sich nicht, auch nicht bei der späteren flexiblen Ösophagogastroduodenoskopie (ÖGD). Die Patientin war kreislaufstabil, die Sauerstoffsättigung lag im Normbereich.

## Diagnose

Es handelte sich um eine vollständige laryngotracheale Separation.

### Therapie und Verlauf

**Figure 3:**
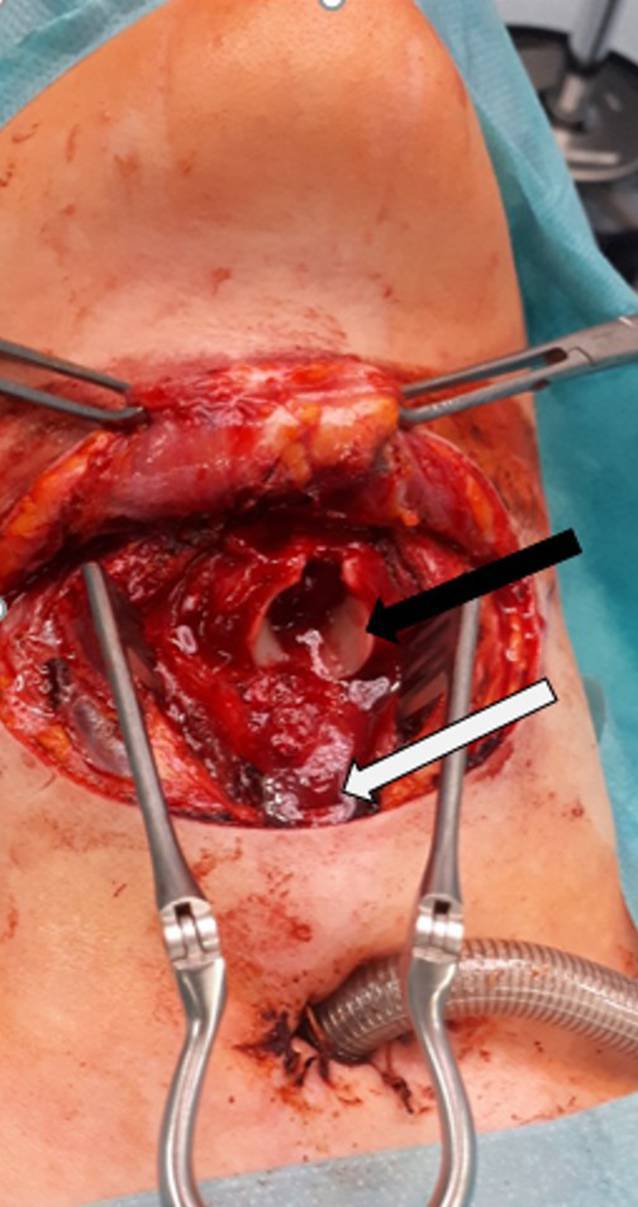

Nach Vorliegen der radiologischen Befunde wurde noch im Schockraum eine Notfalltracheotomie durchgeführt. Endoskopisch ließ sich der Larynx über das Tracheostoma nicht einstellen, das Lumen der Trachea war nach kranial verlegt. Transoral war die Glottisebene gut einstellbar, die Schleimhaut der Arytänoidregion war leicht geschwollen. Unterhalb der Glottis zeigten sich ventral Schleimhautlefzen sowie eine Kontinuitätsunterbrechung. Koagel und kleinere Sickerblutungen sowie die Schleimhautlefzen verhinderten eine genaue Einschätzung des Verletzungsausmaßes, weshalb der Situs von außen exploriert wurde. Es zeigten sich eine Sprengung des Arcus des Ringknorpels in der Mittellinie, multiple Frakturen im Bereich der Lamina des Ringknorpels sowie eine vertikale Fraktur des Schildknorpels in der Mittellinie (Mischbild der Kehlkopfverletzung nach Richter [10]). Der Ringknorpel besaß keine Schleimhautauskleidung mehr. Außerdem bestätigte sich der Verdacht einer vollständigen laryngotrachealen (krikotrachealen) Separation (Grad 5 der Klassifikation der Kelhkopftraumata nach Fuhrmann [6], Abb. 3). Das Lumen der Trachea war von Schleimhautfetzen des Paries membranaceus verlegt, und die Trachea hatte sich in Richtung Mediastinum zurückgezogen.

Aufgrund des komplexen Verletzungsmusters wurde ein zweizeitiges Vorgehen geplant. Im ersten Schritt erfolgten folgende rekonstruktive und stabilisierende Maßnahmen:

- Versorgung der Schildknorpelfraktur,
- Auskleidung des Ringknorpellumens mit Mundschleimhauttransplantaten,
- Rekonstruktion des Paries membranaceus der Trachea mit originärem Gewebe und Mundschleimhaut,
- Einsetzen eines aus der verletzten ersten Trachealspange entnommenen Knorpeltransplantats in die Lücke des aufgesprengten Ringknorpels,
- Fixieren des Trachealstumpfs an der Haut und
- Einlage eines Montgomery-Tubus.

Eine Reanastomosierung in derselben Sitzung erschien insbesondere aufgrund der ausgeprägten Verletzungen des Ringknorpels nicht sinnvoll.

Die funktionelle Untersuchung mittel Videolaryngoskopie und funktioneller endoskopischer Schluckuntersuchung (FEES) ergab eine Dysphagie mit Aspirationsgefahr sowie eine beidseitige Rekurrensparese. Es erfolgte die Anlage eine Magensonde über eine perkutane endoskopische Gastrostomie (PEG).

Nach einem Intervall von 16 Tagen wurde die Trachea stumpf aus dem Mediastinum nach kranial mobilisiert, der Kehlkopf wurde nach kaudal verlagert, und es erfolgte die End-zu-End-Anastomosierung. Das Stoma wurde dabei nach kranial verlagert. Erschwert war die Prozedur durch die schlechte Mobilisierbarkeit der Trachea, die weiterhin vorhandene relative Instabilität des Larynx, eine weiche und brüchige Konsistenz der kranialen Trachealspangen (mit geringem Abstand zum Tracheostoma) sowie durch einen deutlichen Kalibersprung zwischen Ringknorpel und Trachea, der durch das Einsetzen eines Knorpelstücks in den ventral aufgesprengten Ringknorpel entstanden war.

Im weiteren Verlauf kam es wiederholt zu deliranten Phasen. Eine Magnetresonanztomographie des Kopfs (cMRT) ergab keinen Anhalt für postischämische zerebrale Veränderungen. Die delirante Symptomatik bildete sich im Verlauf vollständig zurück.

**Figure 4:**
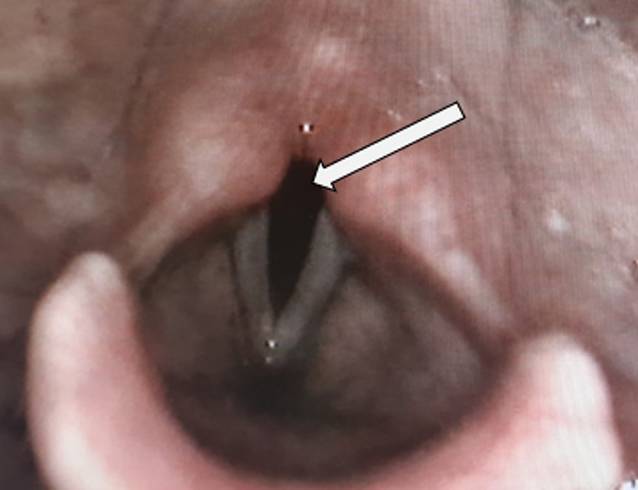

Der Kostaufbau sowie die Verwendung einer Sprechkanüle erfolgten 4 Wochen nach dem letzten operativen Eingriff. Aufgrund der beidseitigen Rekurrensparese war jedoch nur eine sehr verhauchte Phonation möglich. Nach weiteren 2 Wochen war die Ernährung vollständig p.o. möglich. Die Stimmlippen befanden sich in paramedianer bis intermediärer Stellung, sodass ein respiratorisch ausreichend weiter Glottisspalt vorhanden war (Abb. 4). Die Patientin konnte mit abgeklebtem Stoma aus dem Krankenhaus entlassen werden.

## Diskussion

Laryngotracheale Verletzungen sind aufgrund der anatomischen Gegebenheiten (relativer Schutz durch Sternum und Mandibula) selten. Balci et al. (2002) identifizierten unter 8600 Patienten mit Thoraxtrauma lediglich 32 mit einer derartigen Verletzung (0,37 %). Durch den knorpeligen Aufbau, Mobilität und Elastizität entsteht ein zusätzlicher Schutz. Weniger als 1 % aller laryngealen Traumata gehen mit einer laryngotrachealen Separation einher [1, 14]. Kommt es aber zu einer solchen, so ist die Mortalität hoch. Häufig versterben Betroffene, bevor sie die Klinik erreichen [9].

Die Seltenheit dieser Verletzungen sowie die oft unspezifischen und initial wenig spektakulären Symptome können dazu führen, dass die Diagnose verzögert gestellt wird. Ein zügiges und entschlossenes Management ist jedoch für die Prognose entscheidend. Ein vermeintlich stabiler Patient kann in kürzester Zeit in einen lebensgefährlichen Zustand geraten, wenn der Atemweg nicht mehr ausreichend durchgängig ist [3, 15, 16]. Im hier geschilderten Fall konnte die Patientin nach dem Trauma zunächst noch sprechen und sich selbstständig zu Fuß zum Rettungswagen begeben, bevor sich ein ausgeprägter Stridor mit kritischer Dyspnoe entwickelte.

Die Trias aus stattgehabtem Trauma, Dyspnoe/Stridor und Emphysem sollte den Kliniker unbedingt an eine laryngotracheale Verletzung mit potenziell gefährlichem Verlauf denken lassen. Weitere Symptome sind Heiserkeit (die initial auch das einzige Symptom sein kann), Hämoptoe und Hautverletzungen [2]. Wichtigste diagnostische Maßnahmen sind die flexible transnasale Laryngoskopie, die Bronchoskopie sowie die Schnittbildgebung mittels CT. Zusätzlich sollte eine Verletzung der Halsgefäße, der Halswirbelsäule sowie des Ösophagus (ÖGD oder Ösophagusbreischluckuntersuchung) ausgeschlossen werden. Übersichten zur Diagnostik und Therapie bei laryngealen und laryngotrachealen Traumata finden sich bei Ernst et al. [4, 5], Sandhu und Nouraei [11] und bei Schaefer [12].

In der Literatur sind Fälle mit vollständiger laryngotrachealer Separation beschrieben, die von den Betroffenen überlebt wurden [8, 13, 14, 17]. Im hier beschriebenen Fall ist es aufgrund der Befundkonstellation besonders bemerkenswert, dass die Patientin überlebte und keinen hypoxischen Hirnschaden davontrug. Zwar konnte sie am Unfallort intubiert werden, der Tubus lag jedoch nicht korrekt. Außerdem war die Trachea durch Schleimhaut zumindest subtotal verlegt.

Nach laryngotrachealer Separation sollte eine frühzeitige Reanastomosierung erfolgen, insbesondere um subglottischen Stenosen vorzubeugen [7, 8]. Im hier beschriebenen Fall wurde bewusst ein zweizeitiges Vorgehen gewählt, weil der Ringknorpel zu stark geschädigt war und bei der chirurgischen Exploration für eine sofortige Reanastomosierung zu instabil erschien. Zur Vorbeugung einer Ringknorpelstenose wurde ein Knorpelstück in den ventral aufgesprengten Ringknorpel eingesetzt anstatt zu versuchen, die Bruchenden direkt aneinander zu adaptieren. Hiermit wurde – zumindest nach dem aktuellen Stand der Beurteilung – ein weiter subglottischer Durchmesser erreicht. Ein gewisser Nachteil für die spätere Reanastomosierung war jedoch die entstandene Kaliberdifferenz zum Trachealstumpf.

Bei einer laryngotrachealen Separation besteht eine sehr hohe Wahrscheinlichkeit für eine beidseitige Rekurrensschädigung, die nicht selten dauerhaft ist. Nach einer Wartezeit von mindestens 9–12 Monaten lässt sich absehen, ob eine Erholung zu erwarten ist oder nicht. Bis dahin ist eine intensive phoniatrisch-logopädische Weiterbetreuung erforderlich. Im Fall einer bleibenden Parese können glottiserweiternde Eingriffe eine Dekanülierung des Patienten ermöglichen. Im hier gezeigten Fall befanden sich die Stimmlippen nicht in einer klassischen Median‑/Paramedianstellung, sondern in einer paramedianen bis intermediären Stellung, sodass ein relativ weiter Glottisspalt resultierte und die Patientin nach Dekanülierung keine Dyspnoe und keinen Stridor zeigte.

## Fazit für die Praxis

- Bei posttraumatischen Strangulationsmarken, Emphysem, Stridor und Dyspnoe sollte auch bei initial harmlos erscheinender Symptomatik an eine potenziell lebensbedrohliche Verletzung des Kehlkopfs oder der Trachea unterschiedlichen Ausmaßes gedacht werden.
- Bei Verletzungen des Kehlkopfs kann eine initial harmlose Symptomatik rasch zu einer lebensbedrohlichen Situation hin fortschreiten.
- Flexible transnasale Laryngoskopie, Bronchoskopie und Computertomographie sind wichtige diagnostische Tools.
- Darüber hinaus müssen Verletzungen der großen Halsgefäße, der Halswirbelsäule sowie des Ösophagus ausgeschlossen werden.
